# Synthesizing the First Phase of Dynamic Sequences of Breast MRI for Enhanced Lesion Identification

**DOI:** 10.3389/fonc.2021.792516

**Published:** 2021-12-07

**Authors:** Pingping Wang, Pin Nie, Yanli Dang, Lifang Wang, Kaiguo Zhu, Hongyu Wang, Jiawei Wang, Rumei Liu, Jialiang Ren, Jun Feng, Haiming Fan, Jun Yu, Baoying Chen

**Affiliations:** ^1^ Clinical Experimental Centre, Xi’an International Medical Center Hospital, Xi’an, China; ^2^ Imaging Diagnosis and Treatment Center, Xi’an International Medical Center Hospital, Xi’an, China; ^3^ The School of Computer Science and Technology, Xi’an University of Posts and Telecommunications, Xi’an, China; ^4^ GE Healthcare China, Beijing, China; ^5^ The School of Information of Science and Technology, Northwest University, Xi’an, China; ^6^ The School of Medicine, Northwest University, Xi’an, China

**Keywords:** generative adversarial network (GAN), images synthesis, breast cancer, deep learning, magnetic resonance imaging (MRI)

## Abstract

**Objective:**

To develop a deep learning model for synthesizing the first phases of dynamic (FP-Dyn) sequences to supplement the lack of information in unenhanced breast MRI examinations.

**Methods:**

In total, 97 patients with breast MRI images were collected as the training set (n = 45), the validation set (n = 31), and the test set (n = 21), respectively. An enhance border lifelike synthesize (EDLS) model was developed in the training set and used to synthesize the FP-Dyn images from the T1WI images in the validation set. The peak signal-to-noise ratio (PSNR), structural similarity (SSIM), mean square error (MSE) and mean absolute error (MAE) of the synthesized images were measured. Moreover, three radiologists subjectively assessed image quality, respectively. The diagnostic value of the synthesized FP-Dyn sequences was further evaluated in the test set.

**Results:**

The image synthesis performance in the EDLS model was superior to that in conventional models from the results of PSNR, SSIM, MSE, and MAE. Subjective results displayed a remarkable visual consistency between the synthesized and original FP-Dyn images. Moreover, by using a combination of synthesized FP-Dyn sequence and an unenhanced protocol, the sensitivity, specificity, positive predictive value (PPV), and negative predictive value (NPV) of MRI were 100%, 72.73%, 76.92%, and 100%, respectively, which had a similar diagnostic value to full MRI protocols.

**Conclusions:**

The EDLS model could synthesize the realistic FP-Dyn sequence to supplement the lack of enhanced images. Compared with full MRI examinations, it thus provides a new approach for reducing examination time and cost, and avoids the use of contrast agents without influencing diagnostic accuracy.

## Introduction

Breast cancer has become the most frequently-occurring tumor in women with an increasing incidence (1, 2). Breast magnetic resonance imaging (MRI) examinations have high sensitivity for detecting breast cancer (3–5), and have been recommended for the screening of high-risk groups to reduce breast cancer mortality by the American Cancer Society and the European Society of Breast Imaging (6, 7). However, a traditional full MRI examination protocol includes not only plain scanning and diffusion-weighted image (DWI), but also dynamic-contrast enhanced (DCE) sequences. The long image acquisition times, high cost, and the risk of contrast agent allergy have limited its widespread application for breast cancer screening (8, 9). In order to abbreviate total scan time, Kuhl et al. (10) built an abbreviated MRI protocol with equivalent diagnostic accuracy to the traditional full MRI protocol, in which contrast agent is still requested. Thereafter, Baltzer PA et al. (11, 12) proposed an unenhanced abbreviated breast MRI (u-AB-MRI) protocol, including plain scanning and DWI. This protocol can significantly reduce the scanning time and is free of the contrast agent, but the diagnostic performance may be reduced due to the missing DCE sequence images. Thus, it is urgent to obtain DCE sequence images without actual scanning to compensate for the limitations in the u-AB-MRI protocol.

Generative Adversarial Network (GAN) is a deep learning framework which has been applied to image-to-image translation (13, 14). The new techniques based on the GAN framework can contribute to addressing challenging tasks in medical imaging (15, 16), particularly for converting an image from one modality into the other (17–19), such as synthesizing MRI images from CT images (20, 21). However, the breast MRI images have a complex structure and uneven gray distribution. DCE-MRI, on the other hand, has high spatial resolution and can reflect the morphologic and hemodynamic features of breast lesions (22). Thus, it is challenging to synthesize an enhanced MRI image based on a plain scan image using a GAN model. To address these drawbacks, we propose to build an effective novel method, which can precisely learn the nonlinear mapping from MRI plain scan images to enhanced images, to synthesize realistic enhanced images.

In our study, a deep learning model was developed to synthesize the first phases of dynamic (FP-Dyn) sequence in order to compensate for the paucity of information in unenhanced breast MRI examinations. We demonstrated that the synthesized FP-Dyn sequence combined with an unenhanced protocol had a similar diagnostic value to the traditional full MRI examinations. To our knowledge, this is the first study to attempt to obtain breast enhanced MRI images without scanning, and further preliminarily evaluate the diagnostic performance of the synthesized enhanced images. It may provide a new idea to reduce the cost, examination time, and avoid the use of contrast medium in breast MRI, which is conducive to the popularity of breast MRI.

## Materials and Methods

### Patient Population

This study retrospectively collected the breast MRI images from 97 patients who underwent MRI examinations between 2019 and 2021 at Xi’an International Medical Center Hospital, and patients were randomly split into a training set (n = 45), a validation set (n = 31) and a test set (n = 21), respectively. The inclusion criteria for the MRI images were as follows: I) image acquisition at 3.0T magnetic field; II) excellent image quality with no motion artifacts. The exclusion criteria were as follows: I) images with an incomplete sequence; II) incomplete clinical data; and III) a history of surgical resection. This research was approved by the Ethics Committee of the Xi’an International Medical Center Hospital and was conducted according to the principles of the Declaration of Helsinki.

### MRI Protocols

Image acquisition was performed using the MAGNETOM Prisma 3.0T MRI. The imaging protocol mainly included T1-weighted (T1w), T2-weighted (T2w), DWI, and T1w sequences after contrast administration. Here, the Gd-DTPA (0.1 mmol per kilogram body weight) was intravenously injected at an injection rate of 2mL/s, and then the same amount of normal saline was injected. Furthermore, the scan parameters were as follows: Axial T1w: 176 slices, slice thickness = 1.0 mm; FOV =384×384 mm; TR/TE, 5.66/2.46 ms; matrix = 384×384. Axial T2w: 160 slices, slice thickness = 0.9mm; FOV =360×230 mm; TR/TE, 2000/220ms; matrix = 400×256. Axial gadolinium-enhanced T1W: 176 slices, slice thickness = 1.0 mm; FOV = 384×384 mm; TR/TE, 4.66/1.62 ms; matrix = 384×384. Axial DWI: 35 slices, slice thickness = 4.0 mm; TR/TE, 6100/65ms; FOV = 168×340 mm, b = 0 s/mm^2^ and 1,000 s/mm^2^; EPI factor = 84; matrix = 84 ×170; bandwidth = 2262 Hz/pixel.

### Data Preprocessing

We used the following steps for data preprocessing. Firstly, the original T1WI and FP-Dyn images in DICOM format were converted to PNG format by using the MicroDicom Viewing software (http://www.microdicom.com/). Then, TIWI images were subtracted from the original FP-Dyn images, and applied a threshold to obtain the contrast agent enhancing areas. Furthermore, using the FP-Dyn images, the edge detail label was obtained through further Canny edge detection. Moreover, the pixel values for each image were scaled into the range [0,1] by using the min-max scaling method. Finally, we expanded the training datasets through the data augmentation strategies, which included rotating, cropping, and mirroring them to improve the performance and robustness of the model (23, 24).

### Model Architecture

**Figure 1:**
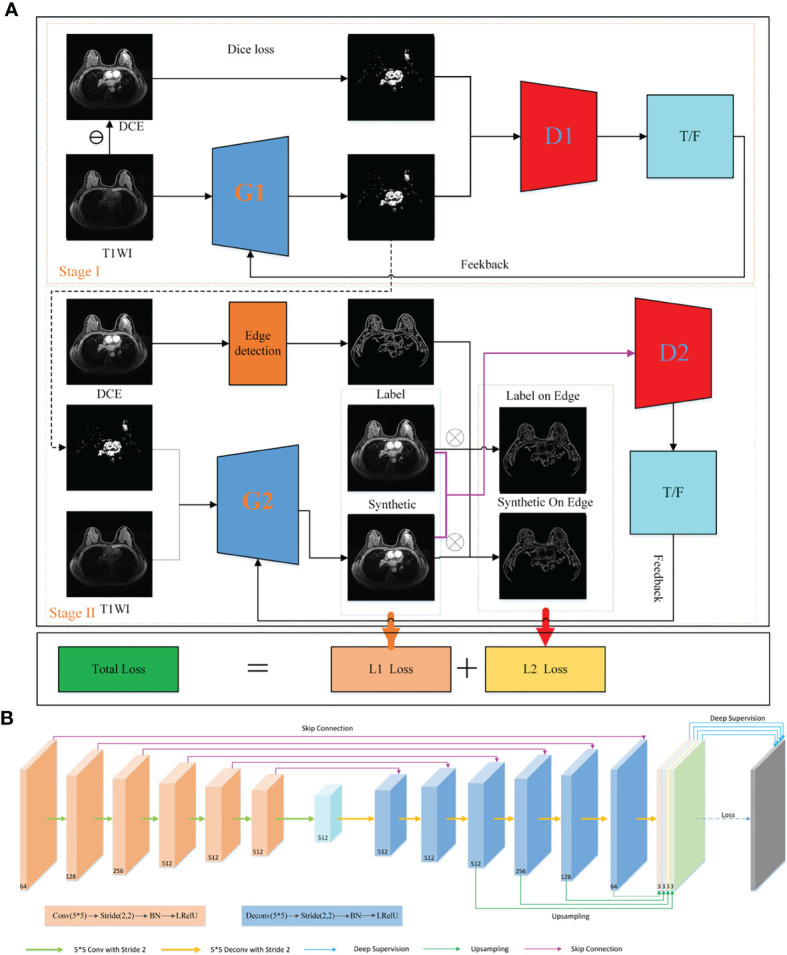
The flowchart for synthesizing FP-Dyn sequences by using EDLS. **(A)** A flowchart for constructing the EDLS model. On stage I, the generator G1 transferred the T1WI image to the images only containing an enhanced area. The D1 discriminator was used to judge the consistency of the synthesized enhanced area image with the original area images. In stage II, synthesized FP-Dyn sequence images were synthesized by a generator G2 from T1WI sequence images and enhanced area images. In addition, the edge loss function was added to ensure the details of the synthesized FP-Dyn sequence image. The loss functions of the EDLS model consist of two parts: L1 Loss and L2 Loss. **(B)** Showed the structure and detailed parameter information of the generators (G1, G2), and the G1 and G2 had similar structures and parameters. The U-Net was applied to the network architecture of generators, and a deep-supervision strategy was used to optimize the training process.

The enhance border lifelike synthesize (EDLS) model consists of two components: a segmentation network (stage I) and a synthesis network (stage II). The two networks have a similar network structure, including a generator using the U-Net network and a discriminator based on the convolutional neural network, respectively. First, the segmentation network was constructed to segment the potentially contrasted agent enhancing areas of T1WI images. Then, the synthesis network was used to produce the FP-Dyn sequence images from the input T1WI images and the segmentation information. Here, the segmentation information was used as additional information to supervise the contrasted agent enhancing areas in the synthetic images (**Figure 1**).

Furthermore, the Dice loss function was used to train the segmentation network in order to tackle the class imbalance problem in the enhanced area and the non-enhanced area. As shown in formula 1.


(1) $$l_{seg}(\Omega )=1-\left(\frac{2\sum_{s\in \Omega }p(s)*y(s)}{\sum_{s\in \Omega }p(s)+\sum_{s\in \Omega }y(s)+\epsilon }\right)$$

Where, Ω indicated the set of pixels in the whole image, *s* represented the pixel, and *p(s)* represented the predicted probability for *s* category by the segmentation network, *y(s)* represented the true category label of pixel *s*, and ε denoted the smoothing coefficient.

In the synthesis network, the edge loss function and depth supervision strategy were introduced to solve the problem of blurring of edges and details in the synthesized image. The detailed information was displayed in formula 2-6.


(2) $$l_{cGAN}(G,D)=E_{x,y}\left[\log D(x,y)\right]+E_{x,x_{seg}}\left[\log(1-D(x,G(x,x_{seg}))\right]$$

(3) $$l_{layer\_L1}(G)=E_{x,y,x_{seg}}\left[||y-G_{layer}(x,x_{seg})|{|}_{1}\right]$$

(4) $$l_{layer\_L2}(G)=E_{x,y,x_{seg}}\left[||y*y\_edge-G_{layer}(x,x_{seg})*y\_edge|{|}_{1}\right]$$

(5) $$l_{DS}=\sum_{layer=c}^{n}\left(\lambda_{1}l_{layer\_L1}+\lambda_{2}l_{layer\_L2}\right)$$

(6) $$l_{adv}=\arg\underset{G}{\min}\underset{D}{\max}(l_{cGAN}(G,D))+l_{DS}$$

The *G* and *D* represented the generator and discriminator respectively. *x* represented the original T1WI sequence images, *x_seg_* was the segmented image corresponding to *x*, *y* was the original FP-Dyn sequence image, *y*_*edge* represented the *y* image edge information map obtained by Canny operator detection, *l_cGAN_* represented the generation countermeasure loss function, and the loss function *l_layer__L*1 was introduced to further ensure the similarity between the synthesized image and the original image. In addition, to solve the problem of lacking edge detail information in the synthesized FP-Dyn images, we added the edge detail loss function *l_layer__L*2 into the model. Furthermore, the depth supervision network *l_DS_* was added into the model for stable convergence. And, the λ_1_ and λ_2_ were weighted coefficients and assigned values of 1 and 6 according to the experimental experience, respectively.

### Model Training and Testing

The EDLS model training task sought to learn the mapping specifically between T1WI sequence images and FP-Dyn sequence images. Here, the EDLS model was trained on a training set including 3996 pairs of T1WI-FP-Dyn images. We optimized the loss functions by using the Adam optimizer during the training stage, and the model parameters were updated for each training iteration until the model converged. In addition, we implemented all the models with Python 3.6. The TensorFlow framework was used for model construction and evaluation. The software and hardware included CUDA9.0, cuDNN7.6.5, and a Linux server with 2 NVIDIA GTX 1080Ti GPUs.

Following the completion of the model training, the EDLS model was used to synthesize the FP-Dyn images and sequences based on 1226 T1WI images and 25 T1WI sequences in the validation set, respectively. Furthermore, we also synthesized the FP-Dyn sequences using the EDLS model for each patient in the test set.

### Conventional GAN Model

We compared the performance of the EDLS model to conventional GAN models, including CycleGAN (25), DC2Anet (26), MR-GAN (27), and Pix2Pix (28). Conventional models were trained and evaluated using the same data set as the EDLS model.

### Quantitative Analysis of Model Performance

The model performance was evaluated by using the quantitative index of peak signal-to-noise ratio (PSNR), structural similarity (SSIM), mean square error (MSE) and mean absolute error (MAE) (29). A lower MSE and MAE, or a higher PSNR and SSIM, indicated a better model performance for image synthesis (30).

### Subjective Evaluation of Image Quality

The test images, including the synthesized FP-Dyn images and the original FP-Dyn images, were randomly put together. Three radiologists independently discriminated between the synthesized and original FP-Dyn images. In addition, with reference to the original image, the satisfaction of the synthesized images was evaluated by the radiologists using a five-point Likert scale (1 disagree strongly, 2 disagree, 3 no response, 4 agree, and 5 agree strongly). Further, we dichotomized the evaluated results. Scores equal to or greater than 4 were defined as “satisfied”. Scores of 1 to 3 were defined as “dissatisfied”.

### Assessment of Diagnostic Value

Three reading modes, including reading mode (a), reading mode (b), and reading mode (c), were designed to evaluate the diagnostic value of the synthesized FP-Dyn sequence. And the reading mode (a) included T1WI, T2WI, DWI, and the synthesized FP-Dyn sequence. The reading mode (b) included the T1WI, T2WI, DWI and the originally scanned FP-Dyn sequence. The reading mode (c) included the T1WI, T2WI, DWI and the total phases of scanned DCE sequences. Then, three experienced breast radiologists independently diagnosed under each of the three modes, respectively. There was a minimum of one month between each of the reading modes. The breast lesions were diagnosed according to the BI-RADS classification. Here, a BI-RADS score greater than 3 was considered positive, while 3 or less was considered negative. We compared the sensitivity, specificity, positive predictive value (PPV), and negative predictive value (NPV) among the afore-mentioned three reading modes, using the pathology results as the gold standard.

### Statistical Analysis

In this study, continuous variables were presented as mean and standard deviation, and categorical variables were presented as percentages. The Analysis of Variance (ANOVA) test was used to analyze the performance differences between our model and the conventional GAN models. In addition, the diagnostic consistency among three different reading modes was calculated from the Kappa test. Furthermore, the Kendall test was used to evaluate the consistency of the diagnoses among the radiologists. A two-side *P*-value less than 0.05 was considered statistically significant. The Holm method was used to adjust the *P* value between multiple group comparisons. All the statistical tests were performed in R (version 3.6.3, https://www.rproject.org) software.

## Results

### The Performance of Models

**Figure 2:**
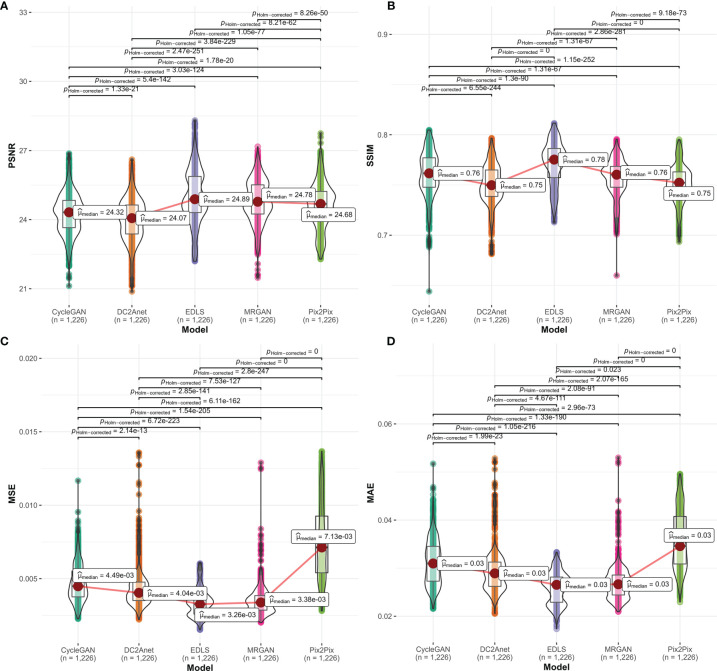
The comparison of the performance between our model and conventional models on **(A)** PSNR, **(B)** SSIM, **(C)** MSE, and **(D)** MAE metrics. From left to right, the violin plots with a median (orange line) respectively represented CycleGAN, DC2Anet, EDLS, MR-GAN, and Pix2Pix.

We quantitatively compared the 1226 FP-Dyn images which were respectively synthesized by the EDLS model and the conventional GAN models. Here, the results which were measured by the MAE, MSE, PSRN, and SSIM were summarized in **Figure 2**. It was easy to see that the performance of the EDLS model was significantly superior to other conventional GAN models. Compared with the Pix2Pix model, the EDLS model produced improvements of more than 2% in SSIM, which played an important role in improving the quality of the synthesized images. Simultaneously, the PSNR had also been significantly improved (*P*<0.001). In addition, we found that the EDLS model had the lowest MAE and MSE (*P*<0.001).

The results of PSNR, SSIM, MAE and MSE from the synthesized FP-Dyn sequences in 25 patients were shown in **Supplementary Figure 1**. The experimental results suggested that the EDLS model had the highest PSNR and SSIM (*P*<0.001), and had the lowest MAE and MSE (*P*<0.001), compared with the conventional models (**Supplementary Figure 1**).

### Visual Evaluation Between the Synthesized and Original FP-Dyn Images

The 1226 synthesized FP-Dyn images and 1226 original FP-Dyn images were randomly put together. As seen in **Table 1**, one radiologist correctly identified images with 52.00% accuracy of 2452 images, and the precision rate for correctly identifying the original FP-Dyn image was 51.20% of 1226 images. In addition, each of the other two radiologists correctly identified images with 56.65% and 53.67% accuracy, and 54.35% and 52.71% precision.

**Table 1:** The results of the visual evaluation between the synthesized and original FP-Dyn images.

Reader	Accuracy	Precision
Doctor1	52.00%	51.20%
Doctor2	56.65%	54.35%
Doctor3	53.67%	52.71%

### Subjective Quality Evaluation of the Synthesized Images

Qualitative metrics for image quality evaluation were shown in **Table 2**. And there were no significant differences in subjective image quality scores among the three radiologists. Compared with the original FP-Dyn images, more than 99% of the synthesized images in the shape consistency were good, and the scores were 4 or 5. In addition, the contrast enhancement for synthesized images was also given high scores by the three radiologists. For example, more than 91% of synthesized FP-Dyn images got good scores of 4 or 5 points for great vessels and heart enhancement. And more than 75% of synthesized FP-Dyn images in gland enhancement got good scores of 4 or 5 points. Furthermore, from the evaluation results, we found that the synthesized images were effective for the suppression of motion artifacts.

**Table 2:** The satisfaction results of the subjective scoring of synthesized FP-Dyn images.

Reader	Satisfaction Scores			
	Shape consistency	Great vessels and heart enhancement	Gland enhancement	Artifact suppression
Reader 1	1221(99.59%)	1140(92.99%)	968(78.96%)	1195(97.47%)
Reader 2	1224(99.84%)	1119(91.27%)	947(77.24%)	1203(98.12%)
Reader 3	1224(99.84%)	1147(93.56%)	922(75.20%)	1207(98.45%)
F	2.005	5.058	4.905	3.131
*P-value*	0.367	0.080	0.086	0.209

**Figure 3:**
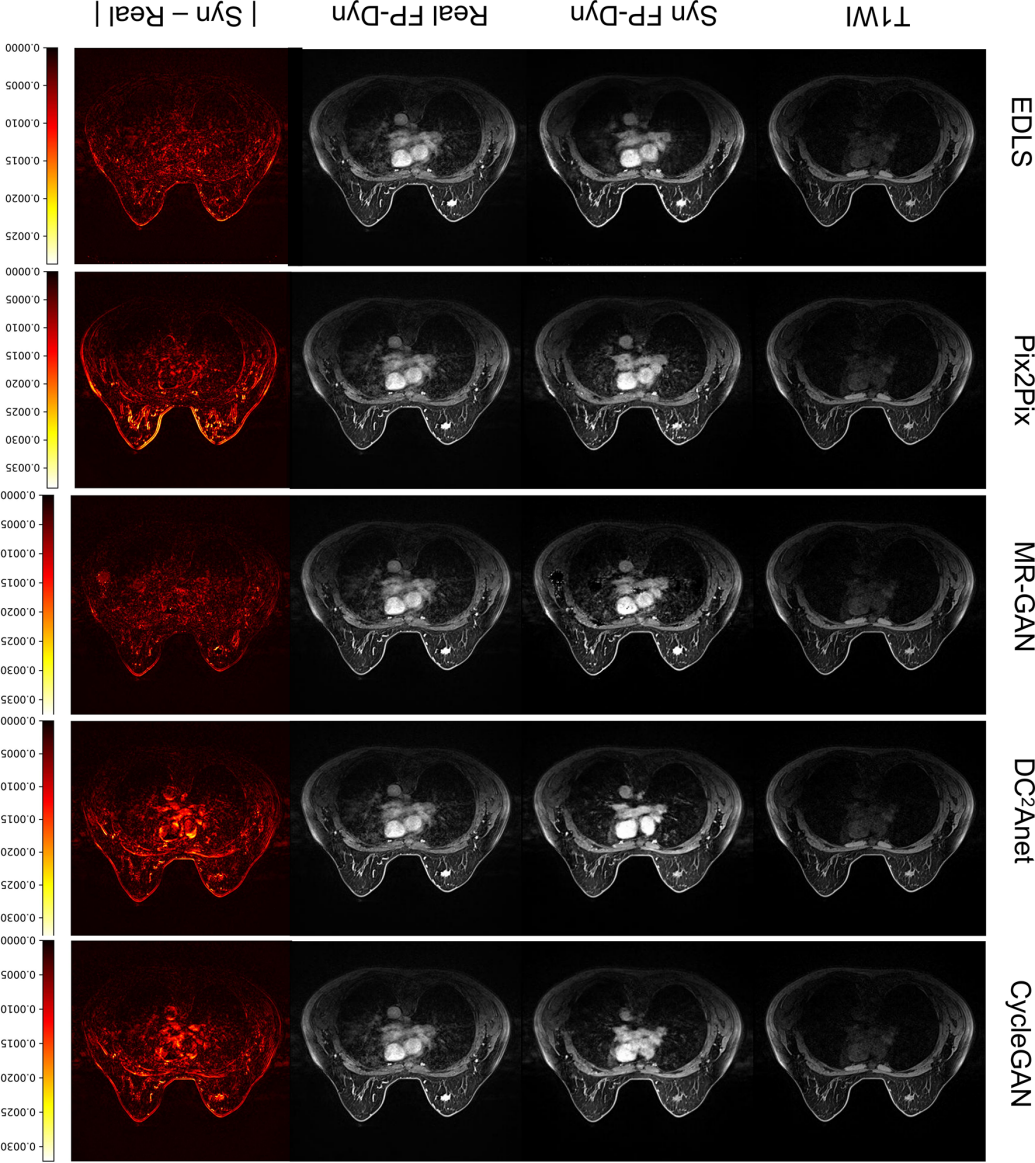
Sample images of the T1WI images, synthesized FP-Dyn images, original FP-Dyn images, and absolute error images. From left to right: T1WI breast MR images, synthesized FP-Dyn images, original FP-Dyn breast MRI, and absolute error images.

In addition, **Figure 3** showed an example of the input T1WI images, synthesized FP-Dyn images, original FP-Dyn images, and absolute error images. The absolute error images showed the absolute error between the synthesized FP-Dyn image and the original FP-Dyn image. We could clearly see that the EDLS model had been learning to identify tissues that had similar signal values in T1WI sequences, but had high signals in FP-Dyn sequences, such as tumor tissue, the heart, and great blood vessels. And the FP-Dyn image synthesized by the EDLS model was highly consistent with the original FP-Dyn image and had a similar enhancement in the lesion. However, the images synthesized by the CycleGAN model failed to effectively enhance the lesions. In addition, there were high background pixel errors from the FP-Dyn images which were synthesized by other models, compared with their original images.

### The Reconstruction Pixel Error of Synthesized Sequence

**Figure 4:**
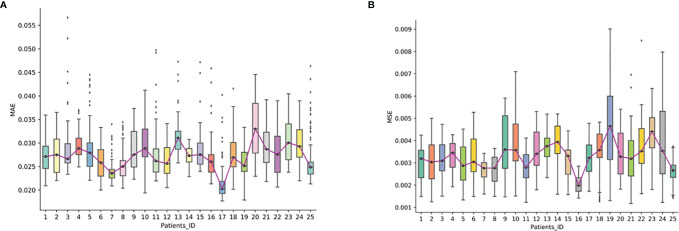
The reconstruction error in synthesized FP-Dyn sequence images. The box plot displayed the data distribution of MAE and MSE of a reconstructed image of the 25 patients, and each of the box plots displayed the data distribution of one patient. **(A)** displayed the data distribution of MAE of a reconstructed image of the 25 patients, **(B)** displayed the data distribution of MAE and MSE of a reconstructed image of the 25 patients.

We further evaluated the reconstruction error of the synthesized FP-Dyn sequence on the validation set. From **Figure 4**, we could see that the synthesized FP-Dyn sequence images had a small reconstruction error compared with the original FP-Dyn sequence images. The average MAE on all sequences was 0.027 with a standard deviation of 0.004, and the average MSE on all sequences was 0.003 with a standard deviation of 0.001. In addition, some outliers of reconstruction errors were detected in the statistical boxplot. And we found there were some gland enhancements in the synthesized FP-Dyn images lower than in the original FP-Dyn images. It might be the major reason for the outliers of reconstruction errors in the boxplot.

In addition, we assessed the correlation between the synthesized FP-Dyn sequences and the original FP-Dyn sequences for each patient in the validation set, then an overall average for sequence correlations was calculated. Here, we found that there were high positive correlations between the synthesized FP-Dyn sequences and the original FP-Dyn sequences. The overall average of the correlations between the synthesized FP-Dyn sequences and the original FP-Dyn sequences was *r* = 0.927 ± 0.311 (95% CI: 0.927 to 0.928, *P* <0.001), (**Supplementary Figure 2**).

### The Diagnostic Value of Synthesized Sequence

We collected a test set of 21 patients who underwent breast MRI examinations and with pathological examination results. Of 11 cases that were diagnosed as benign tumors, 1 case of chronic mastitis, 2 cases of benign epithelial hyperplasia, 2 cases of adenoids, 1 case of fibroadenoma, 1 case of fibroadenoma with adenopathy, 2 cases of fibroadenoma with benign epithelial hyperplasia, and 2 cases of invasive ductal carcinoma without cancer cells in pathology after radiotherapy. The remaining 10 patients had malignant lesions. Of 10 cases that were diagnosed as malignant lesions, 7 cases were non-specific invasive ductal carcinoma, 1 case was non-specific invasive ductal carcinoma with necrosis, and 2 cases were metastatic breast ductal carcinoma. Here, the patients were respectively diagnosed by three experienced breast radiologists using the three-reading mode. The three radiologists had high diagnosis consistency as measured by the Kappa test (Kappa = 0.688, *P*<0.001).

Based on pathology results, we counted the true-positive (TP), false-negative (FN), false-positive (FP), and true-negative (TN) values diagnosed by the three models, and then the sensitivity, specificity, PPV, and NPV values for each reading mode were then calculated, which were listed in **Table 3**. The sensitivity, specificity, PPV, and NPV of the three reading modes for breast cancer diagnosis were as follows. The reading mode (a) was 100%, 72.73%, 76.92%, and 100%, respectively. The reading mode (b) was 100%, 63.64%, 71.43%, 100%, respectively. The reading mode (c) was 100%, 72.73%, 76.92%, and 100%, respectively (**Table 3**).

**Table 3:** The diagnostic values of the synthesized FP-Dyn sequences for breast lesions.

Reading Mode	TP	FP	FN	TN	sensitivity	specificity	PPV	NPV
Mode (a)	10	3	0	8	100%	72.73%	76.92%	100%
Mode (b)	10	4	0	7	100%	63.64%	71.43%	100%
Mode (c)	10	3	0	8	100%	72.73%	76.92%	100%

Importantly, the reading mode (a) and reading mode (b) had the same diagnosis in 20 cases, accounting for 95.24%. The reading mode (a) and reading mode (c) had the same diagnosis results in 19 cases, which accounted for 90.48%. Each of the breast cancer cases both obtained a diagnosis with high BI-RADS scores (4 or 5) by three reading modes, respectively.

## Discussion

In this study, we have developed a novel deep learning model, namely EDLS, for synthesizing the FP-Dyn sequence images, which aims to supplement the lack of enhanced images for breast MRI examinations without contrast agents, and then to ensure diagnostic accuracy, lower scanning time, less cost and higher security. The performance of the EDLS model has been verified and proved to be powerful by using two independent data sets, respectively. This result indicates that the EDLS could synthesize high-quality FP-Dyn sequence images, surpassing conventional GAN models. Of note, compared with the conventional models, the lesion enhancement information, edge loss function, and deep-supervision strategies are added to the EDLS model frame, which may be a major reason for the improvement of synthesized FP-Dyn images.

Notably, we found that the radiologists failed to effectively discriminate between the original FP-Dyn image and the synthesized FP-Dyn image because those images were visually similar. Moreover, the subjective evaluation results by the radiologists demonstrated that the synthesized images had similar details to the original images. And there was a low reconstruction pixel error of synthesized FP-Dyn sequence images. Based on those results, it can be concluded that the EDLS model could synthesize a realistic FP-Dyn sequence, which may be useful to compensate for the diagnosis information paucity in unenhanced MRI examinations.

The diagnostic value of synthesized FP-Dyn sequences should be more considered. Here, the patients were diagnosed by three reading modes, respectively, where reading mode (a) included T1WI, T2WI, DWI and the synthesized FP-Dyn sequence, mode (b) included T1WI, T2WI, DWI, and the originally scanned FP-Dyn sequence, and reading mode (c) included T1WI, T2WI, DWI, and the total phase of the scanned DCE sequences. Meaningfully, the specificity and PPV of the reading mode (a) was 72.73%, 76.92%, and was 63.64%, and 71.43% in the reading mode (b). The reading mode (a) had higher specificity and PPV than the reading mode (b). And it was important to note that with the reading mode (a), breast cancer diagnostic sensitivity, specificity, PPV, and NPV were achieved at equivalent levels to those of the reading mode (c). The results indicate that the synthesized FP-Dyn sequences have diagnostic value for breast lesions.

On one hand, synthesizing the FP-Dyn sequences is critical not only for reducing scanning time and cost, but also for effectively supplementing the lack of information in u-AB-MRI and avoiding the adverse reactions of contrast agents. We have demonstrated that the FP-Dyn sequences can be obtained by the EDLS model without the actual scan. According to our statistics, this MRI protocol acquisition time was substantially 10 to 15 minutes, compared with 25 to 30 minutes for the actual enhanced abbreviated MRI protocol, which can effectively reduce the scan time. A shorter scan time makes it easier for patients to undergo MRI examinations. Furthermore, intravenous access is not required in this MRI protocol, which reduces the cost and avoids the adverse reactions of contrast agents, compared with the full MRI protocol. In addition, the synthesized FP-Dyn sequence images exhibit high similarity to the original images and have diagnostic value for breast lesions, which can effectively supplement the lack of information in u-AB-MRI to ensure the breast MRI diagnosis accuracy. On the other hand, we found the synthesized FP-Dyn images had fewer respiratory motion artifacts than scanning images, which are caused by breathing, movement, and heartbeat that are inevitable during MRI scanning (31, 32). Similarly, Enhao Gong et al. (33) demonstrated that motion artifacts have been reduced in synthetic brain MRI images. Therefore, this method could also help to reduce image motion artifacts to improve the quality of MRI images. Besides, in future work, we plan to integrate this model into an existing platform for medical image processing, which can effectively save deployment and maintenance costs.

There were still several limitations to the current study. First, breast cancer is highly heterogeneous (34, 35), and has different imaging manifestations in different molecular types (36). In this study, we merely collected a relatively small dataset from the MAGNETOM Prisma 3.0T MRI scanner to train and test the EDLS model. Furthermore, in our study, an automatic threshold segmentation algorithm was used to binarize the subtracted T1WI-FP-Dyn MRI images to obtain a rough lesion label. Finally, the clinical application value of the EDLS model should be further verified in a prospective study. Meaningfully, we observed that the sensitivity and specificity for diagnosis were similar between synthesized and original FP-Dyn sequences. Thus, we will expand and collect data from multi-centers to improve the accuracy and universality of the model in the future. Meanwhile, the model will be retrained by the multiparametric sequences (T1WI, T2WI, DWI) to synthesize the DCE sequence images. Also, we plan to manually segment the lesion to ensure the accuracy of lesion enhancement. Most importantly, we will conduct a multi-center, prospective study to verify the clinical application value.

In summary, we proposed a novel deep learning model, i.e., the EDLS model, to synthesize FP-Dyn sequence images. We verified that the EDLS model could synthesize the realistic FP-Dyn sequence. Furthermore, the synthesized FP-Dyn sequence combined with an unenhanced protocol demonstrated a similar diagnostic value to the traditional full MRI examination. Compared with full MRI examinations, it may provide a promising idea to compensate for the paucity of information in unenhanced breast MRI examinations, and reduce cost and scanning time, while avoiding contrast agent allergy.

## Data Availability Statement

The raw data supporting the conclusions of this article will be made available by the authors, without undue reservation.

## Ethics Statement

The studies involving human participants were reviewed and approved by the Ethics Committee of the Xi’an International Medical Center Hospital. The patients/participants provided their written informed consent to participate in this study. Written informed consent was obtained from the individual(s) for the publication of any potentially identifiable images or data included in this article.

## Author Contributions

BC and JY designed this study. PW and KZ collected the image data. PW and HW developed the EDLS model and the conventional GAN model. PN, YD, and LW carried out subjective assessments of synthesized FP-Dyn image quality and the diagnosis value. JW, RL, and PN independently discriminated between the synthesized and original FP-Dyn images. JR, JF, and HF contributed to data analysis. All authors contributed to manuscript writing and editing, and approved the final version of the manuscript for submission. In addition, all authors agree to be accountable for the content of the work.

## Funding

This work was supported by the Key Research and Development Program of Shaanxi Province, the General Project of Social Development (2020SF-049), and the Xi’an Science and Technology Plan Project [20YXYJ0010 (5)].

## Conflict of Interest

Author JR was employed by GE Healthcare China.

The remaining authors declare that the research was conducted in the absence of any commercial or financial relationships that could be construed as a potential conflict of interest.

## Publisher’s Note

All claims expressed in this article are solely those of the authors and do not necessarily represent those of their affiliated organizations, or those of the publisher, the editors and the reviewers. Any product that may be evaluated in this article, or claim that may be made by its manufacturer, is not guaranteed or endorsed by the publisher.
