# Supplementary material for: Synthesizing the First Phase of Dynamic Sequences of Breast MRI for Enhanced Lesion Identification
Source: Front Oncol. 2021 Dec 7;11:792516. doi: 10.3389/fonc.2021.792516 (PMC8689139; doi:10.3389/fonc.2021.792516)
Supplement: Supplementary file 1 [file Image_1.pdf]

Supplementary Material

1 Supplementary Figures

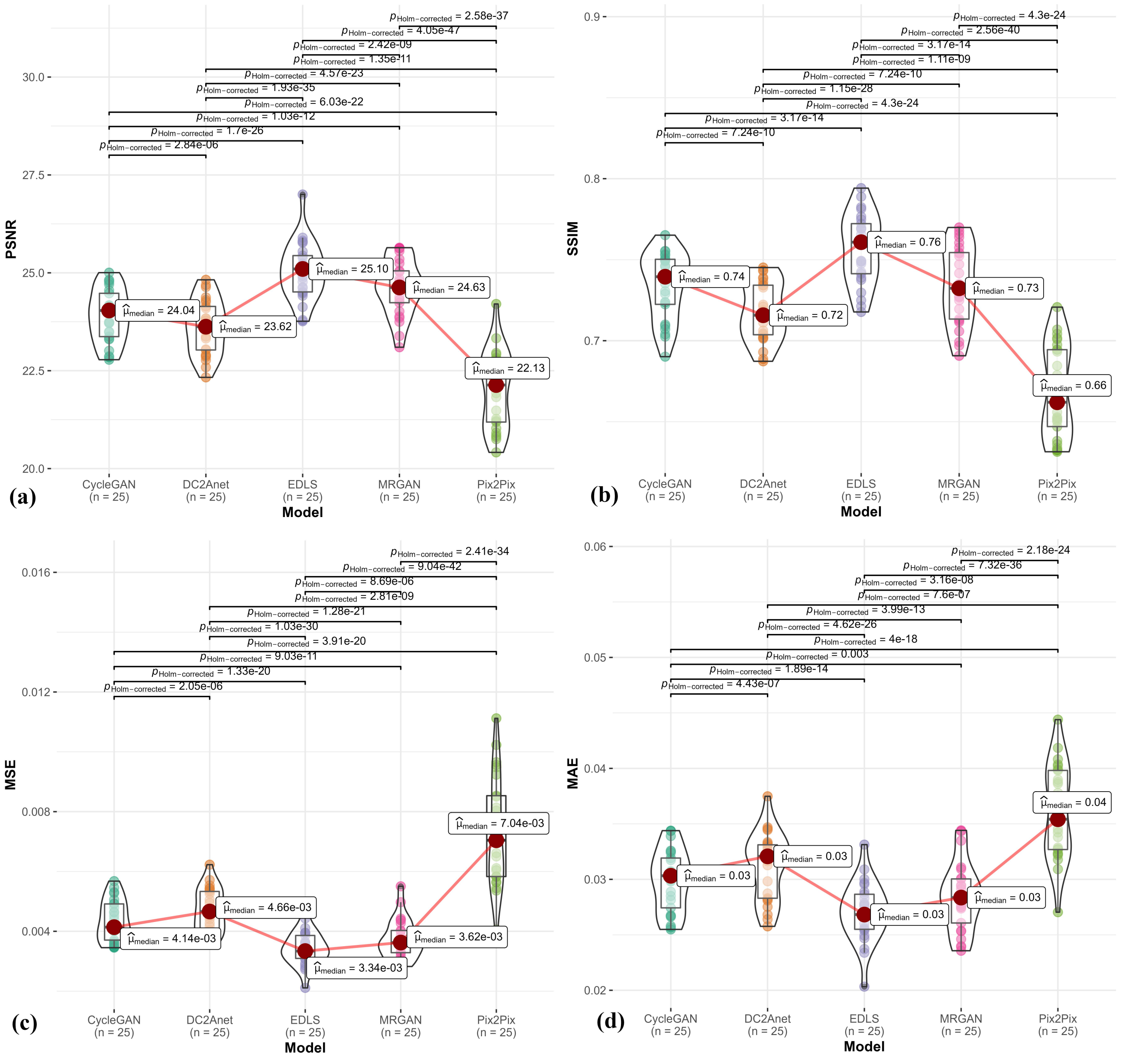

Supplementary Figure 1. The qualitative comparison of the synthesized FP-Dyn sequences from our model and conventional models on (a) PSNR, (b) SSIM, (c) MSE, (d) MAE metrics. From left to right, the violin plots with a median (orange line) respectively represented CycleGAN, DC2Anet, EDLS, MR-GAN, and Pix2Pix.

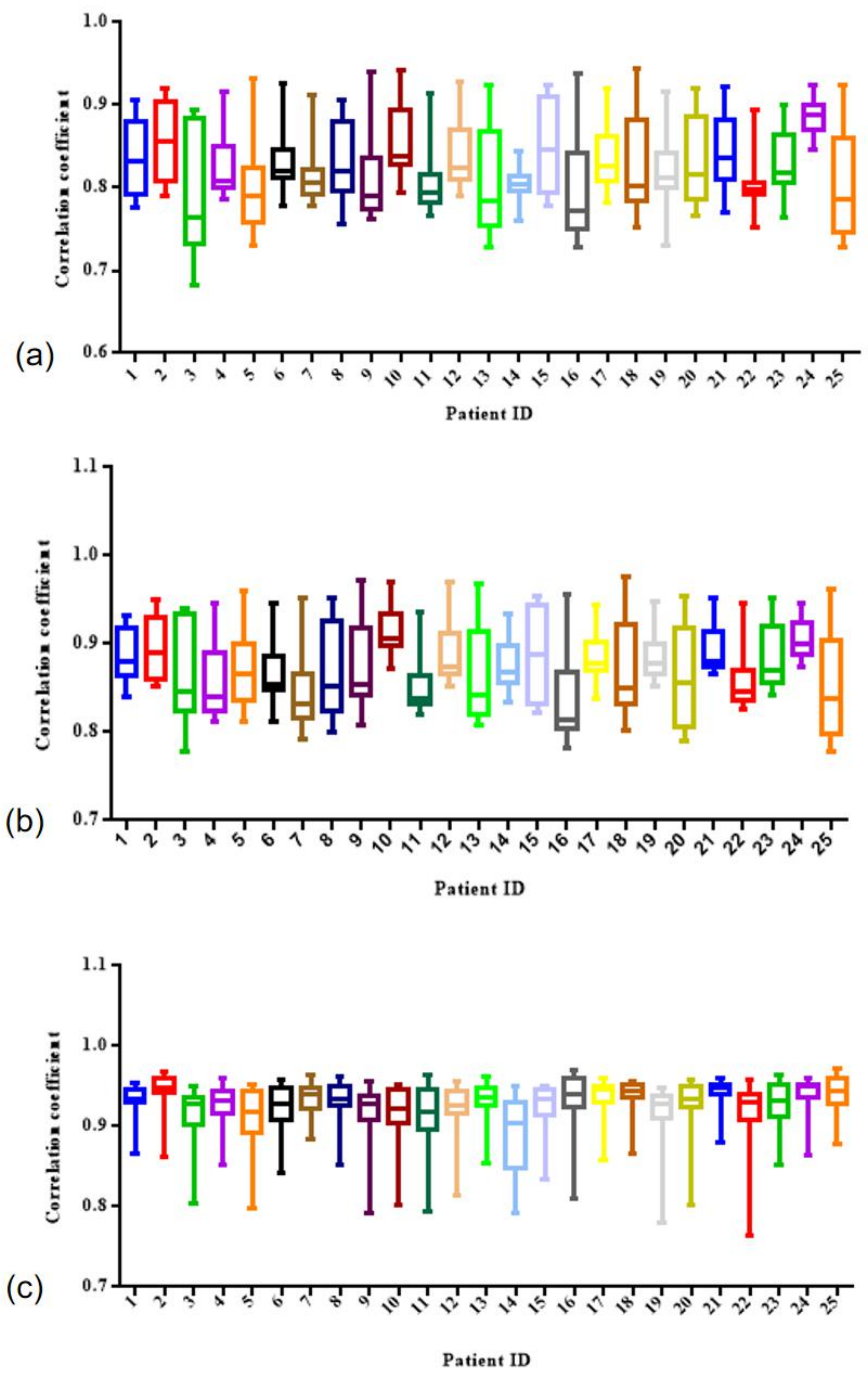

Supplementary Figure 2. The results of the correlation analysis between the synthesized FP-Dyn sequences and the real scan sequences. **(a)** Showed the correlation between the T1WI sequences and the original FP-Dyn sequences in 25 patients, and found that there was a high correlation between T1WI sequences and the original FP-Dyn sequences (r = 0.824±0.051, 95% CI: 0.823 to 0.826). **(b)** There was also a similar significant correlation between the synthesized FP-Dyn sequences and the original T1WI sequences (r = 0.873 ± 0.045, 95% CI: 0.871 to 0.874). **(c)** The average correlation was r = 0. 927±0. 311 (95% CI: 0.927 to 0.928) between the original FP-Dyn sequences and the synthesized FP-Dyn sequences.
